# A systematic review of adult admissions to ICUs related to adverse drug events

**DOI:** 10.1186/s13054-014-0643-5

**Published:** 2014-11-25

**Authors:** Pierre-Alain Jolivot, Patrick Hindlet, Claire Pichereau, Christine Fernandez, Eric Maury, Bertrand Guidet, Gilles Hejblum

**Affiliations:** Institut Pierre Louis d’Epidémiologie et de Santé Publique, Sorbonne Universités, UPMC Univ Paris 06, UMR_S 1136, F-75013 Paris, France; Institut Pierre Louis d’Epidémiologie et de Santé Publique, INSERM, UMR_S 1136, F-75013 Paris, France; AP-HP, Hôpital Saint-Antoine, Service de Pharmacie, F-75012 Paris, France; Univ Paris-Sud, Faculté de Pharmacie, F-92296 Chatenay-Malabry, France; AP-HP, Hôpital Saint-Antoine, Service de Réanimation, F-75012 Paris, France; AP-HP, Hôpital Saint-Antoine, Unité de Santé Publique, F-75012 Paris, France

## Abstract

Adverse drug events (ADE) may lead to hospital admission, and in some cases admission to an ICU is mandatory. We conducted a systematic review dealing with the incidence of ADE requiring ICU admission in adult patients, the reference population being all ICU admissions. Medline, Embase and Web of Science databases were screened from January 1982 to July 2014, using appropriate key words. Only original articles in English reporting the incidence of ADE requiring ICU admission in adult patients among total ICU admissions were included. Article eligibility was assessed by two independent reviewers, a third being involved in cases of disagreement. All reported characteristics (type of ICU, characteristics of patients, incidence of ADE, severity and preventability, drugs involved, causality) in the selected articles were collected for the review. The quality of studies was independently assessed by two reviewers with a specific score that we developed. A meta-analysis was conducted. Inclusion criteria were fulfilled by 11 studies out of the 4,311 identified in the initial literature search. The median (interquartile) quality score was 0.61 (0.44; 0.69). The reported incidences of ADE requiring ICU admission in adult patients ranged from 0.37 to 27.4%, with an associated mortality rate ranging from 2 to 28.1% and a mean length of stay ranging from 2.3 to 6.4 days. Preventable events accounted for 17.5 to 85.7% of the events. Costs and mechanisms at the root of ADE were investigated in only two and five studies, respectively. The forest plot examining the incidence of ADE requiring ICU admission in adult patients was associated with high heterogeneity (*I*^2^ statistic >98%), and the shape of the corresponding funnel plot was asymmetric. Heterogeneity across studies concerned many features, including studied populations, events considered, causality assessment methods, definitions of preventability and severity. Despite the heterogeneity of the reports, our review indicates that ICU admission due to ADE is a significant issue that should deserve further interest. The review led us to propose a list of items devoted to the reporting of future studies on ADE requiring ICU admissions.

**Electronic supplementary material:**

The online version of this article (doi:10.1186/s13054-014-0643-5) contains supplementary material, which is available to authorized users.

## Introduction

The incidence of hospital admissions required because of adverse drug events (ADE) has been reported to range from 0.16 to 15.7% with an overall median of 5.3% [1]. Some serious ADE may require ICU admission. Such unplanned admissions may have consequences for the healthcare system because they may deleteriously overload the ICU and restrain ICU access to other patients. Since ICU admissions generate high costs, with per-capita expenditures ranging from US$730 to 7,410 [2], while the need for critical care capacity is increasing [3], a just and effective rationing of critical care is an issue that all western industrialised countries have to consider. The abovementioned context suggests documenting the incidence of medication-related ICU admissions and the drug management problem involved. Identifying specific patterns in the population of patients admitted to the ICU for ADE – for example, in terms of mortality during ICU stay or after discharge – also constitutes an attractive issue. To gain more insight into all of these issues, we performed a systematic review of the literature on ICU admissions required because of ADE. In this review, we adopted an ICU perspective: the reference with which ICU admissions required because of ADE were contrasted had to be all ICU admissions (not the population of hospitalised patients, for example). In the following text, the incidence of drug-related ICU admissions (IDRIA) will be referred to as the ratio of the number of ICU admissions required because of ADE to the total number of ICU admissions.

As a preliminary, some issues on definitions related to ADE have to be tackled. The terminology of events related to drug harm is complex because many terms have been used in the literature [4]. A definition of ADE was proposed by the Institute of Medicine and was recommended by Nebeker and colleagues [4]: ‘any injury resulting from medical intervention related to a drug’. This broad definition includes both harm caused by the drug itself (that is, adverse drug reaction (ADR), or harm related to the accumulation of drugs in the case of renal or liver failure) and harm resulting from the use of a drug (that is, medication errors). According to the World Health Organization, an ADR is defined as any noxious, unintended or undesired effect of a drug occurring at dosages administered in humans for prophylaxis, diagnosis or treatment [5]. A medication error is ‘any preventable event that may cause or lead to inappropriate medication or patient harm while the medication is in control of health care professional, patient or consumer’ [4]. An iatrogenic event (also called an adverse event) will be considered as ‘any injury related to medical management, in contrast to complications of disease. Medical management includes all aspects of care, including diagnostic and treatment, failure to diagnose or treat, and the systems and equipment used to deliver care’ [6].

## Methods

### Eligibility criteria

The eligibility criteria for inclusion in the review included: original articles in English (reviews, conference abstracts and case reports were excluded); a patient population over 14 years old; hospitalisation in any type of adult ICU (for example, medical, surgical, cardiac, and so forth); main outcome focusing on the ADE as a cause of admission to the ICU, whatever the patients’ origin (home, emergency department, hospital ward), the reference population being all ICU admissions; articles included a description of the incidence of all ICU admissions due to ADE, the reference denominator being the total number of ICU admissions; and articles focusing on admissions to the ICU only due to self-poisoning were excluded.

### Information sources

On 4 July 2014 we conducted a combined search in the Embase database, in the Medline database via PubMed and in the Web of Science. Using relevant search terms related to ADE responsible for admissions to the ICU, we searched for all publications in English from 1 January 1982 to 4 July 2014. Complementary searches were made to identify potential additional articles: the reference lists of retrieved articles as well as their citing lists (the latter being issued from the Web of Science) were hand-searched.

### Search strategy

The queries made in the PubMed, Embase and Web of Science databases are detailed in Additional file 1.

### Study selection

The eligibility of each retrieved article was independently assessed by two reviewers (PAJ and PH) on the title, abstract and, if necessary, full text. We *a priori* decided that in the case of disagreement, a third reviewer (GH) would decide whether to include the study or not.

### Data collection process

A standardised data collection sheet was elaborated by three reviewers (PAJ, PH and GH). One reviewer (PAJ) fulfilled this data collection sheet and another (PH) checked the extracted data. In case of disagreements, the third reviewer (GH) would decide.

### Data items

The list of data items collected in each study is presented in Table 1. Examination of all features reported was used for proposing a list of items that we judged to deserve a mention in articles related to ADE requiring ICU admissions. We redefined the numerous terms related to ADE mentioned in the selected articles using only three terms, namely ADE, ADR and medication errors (defined in Introduction). All costs were normalised in 2014 US dollars and were discounted at an annual rate of 3%.

**Table 1:** **Data items collected for the review**

• Item
• First author^a^
• Year of publication^a^
• Country^a^
• Study design^a^
• Length of study^a^
• Study population^a^
• Inclusion and exclusion criteria^a^
• Definitions of adverse drug events/adverse drug reactions/iatrogenic disease/iatrogenic event^a^
• Definition and/or criteria for causality assessment method^a^
• Definition of severity^a^
• Definition of preventability^a^
• Definition of predictability^a^
• Statistical analyses^a^
• Population characteristics^b^
• Incidence of adverse drug events^b^
• Drugs implied^b^
• Causality results^b^
• Items required for causality assessment methods^b^
• Severity aspects^b^
• Predictability^b^
• Clinical features of adverse drug events^b^
• Length of stay in the ICU^b^
• Costs of hospitalisation in the ICU^b^

^a^General and methodological items. ^b^Items related to Results.

### Risk of bias assessment

To assess the risks of bias in individual studies, a specific list of items adapted to the scope of ADE responsible for ICU admission was designed by three authors (PAJ, PH and CP). The list was based on a combination of Strengthening the Reporting of Observational Studies in Epidemiology (STROBE) [7] and Preferred Reporting Items for Systematic Reviews and Meta-Analyses (PRISMA) [8] items. Two of us (PAJ and PH) independently assessed each individual study by granting for each item either 1 point when reporting of corresponding data was complete or 0 points when corresponding data were missing or reporting was incomplete (disagreements were solved by consensus discussion). Scores per study (proportion of items completely reported) were considered for assessing the risk of bias in individual studies. Scores per item (proportion of studies with the item completely reported) were considered for assessing the risk of bias across studies (risk of bias decreases with increasing score values), as was a funnel plot representing study size against the incidence of ADE. The symmetry of the plot was visually assessed.

### Summary measures

The main measure of our study was the IDRIA. The IDRIA estimate was either directly extracted from the source document or was calculated. The same process was used for obtaining the preventability rate.

### Synthesis of results

Funnel and forest plot analyses were conducted, and risk of bias and heterogeneity across studies were investigated. All analyses were performed using the meta package within R statistical software [9].

### Additional analysis

#### Quality assessment

Risk of bias and quality of reporting are two different notions. We assessed the quality of reporting in the selected articles using a score based on the STROBE checklist; that is, on the quality of reporting items that should be addressed in reports of observational studies [7]. If information on a given item was completely reported in the studied article, 1 point was granted; 0 points were attributed if this information was incomplete or missing. Whenever an item was not applicable for a study, the item was not considered for calculating the study’s score (that is, number of points granted/number of points considered).

In addition, to propose a more specific assessment of the quality in studies reporting the IDRIA, we designed a specific checklist (Table 2) derived from the STROBE checklist, and the quality of reporting of the studies based on this checklist was assessed. Item scores were weighted according to our feeling about item importance, with a highest number of points (3 points) attributed to risk of bias items, and with a binary decision for each item (that is, no grading: either all points associated with the item score granted, or none). For each article, the sum of all points was calculated and the total score was defined as the ratio of the number of points granted to the maximum possible score (excluding nonapplicable items). Quality appraisal therefore varied from 0 to 1, with a score increase corresponding to a quality increase. Scoring was independently assessed by two reviewers (PAJ and PH), and we *a priori* decided that in a case of disagreement a third reviewer (GH) would decide whether to grant the point(s) on a given item.

**Table 2:** **Quality assessment according to specific criteria on adverse drug events responsible for ICU admission**

**Item**	**Recommendation**	**Points granted**
**Title**		
Explicit topic	One must understand that the article concerns ADE that require ICU admissions	2
**Abstract**		
Type(s) of ICU studied	Describe the type of ICU (for example, medical, surgical)	2
Causality assessment method(s)	Indicate the causality assessment method(s) used	2
Proportion of patients admitted to the ICU for ADE	The denominator should be the total number of patients admitted in the studied ICU during the observation period	2
Preventability rate of ADE	Indicate the estimated preventability rate of ADE	1
Study duration	Indicate the study observation period(s)	1
**Introduction**		
Background	Explain the scientific background and rationale for the investigation being reported	2
Objectives	State the objectives of the study	2
**Methods**		
*Mandatory*		
Description of the study design	A prospective patient screening is preferred in order to avoid missing data	3
Type(s) of ICU studied	Describe the type of ICU(s) (for example, medical, surgical)	3
Complementary information on the setting environment	Indicate the presence of eventual other ICU(s) in the hospital, and mention specific wards (oncology, haematology, geriatrics). This information may help in appraising and understanding results	3
Description of the study size rationale	Study size should be argued	3
Definition of ADE	The definition of the institute of medicine and recommended by Nebeker and colleagues [4] should be preferred	3
Evaluation of inter-rater reliability for inclusion decision	Indicate how inter-rater reliability for inclusion decision was assessed	3
Description of evaluators’ training	Describe the profession of evaluators and, if applicable, participation in specific training for the study	3
Description of patients’ screening	Describe who was in charge of the patients’ screening and how screening was performed	3
Description of inclusion/exclusion criteria	Describe and justify inclusion and exclusion criteria	3
Description of collected data and outcomes measured	Collected data should include characteristics of study participants (age, gender, severity score at admission (SOFA/SAPS II), number and classes of drug(s) involved; see items in Results)	3
Description of drug history collecting method	Describe the sources of data used for establishing drug history, including all patients’ prescriptions (home, hospital). If possible, the patient or a relative should be questioned to identify all drugs prescribed, all drugs taken in self-medication and drugs prescribed but not taken (inobservance) during the month prior to ICU admission. If patients were already hospitalised before ICU admission, all drugs administered during the hospital stay should be collected	3
Description of causality assessment method(s)	Mention the causality assessment method(s) used. Assessment of inter-rater reliability would be welcome	3
Description of preventability method/criteria	Mention the criteria used for assessing preventability. Assessment of inter-rater reliability would be welcome	3
Definition of the severity	Mention the severity of the ADE: fatal (ADE contributed to death), life-threatening (ADE requiring organ supply) and moderate (ADE only requiring monitoring)	3
Study duration	Mention the date of beginning and ending of the study	3
*Not mandatory*		
Description of statistical analysis (if applicable)	Describe all statistical methods, if applicable	0.5
Research of medical causes that contributed to ADE (for preventable drug events)	Indicate how the medical causes that contributed to ADE were investigated (that is, drug interactions, contraindications between drugs and patient’s disease, nonappropriate dosage)	0.5
**Results**		
*Mandatory*		
Proportion of patients admitted to the ICU for ADE according to the chosen denominator	The chosen denominator should be the total number of included patients admitted to the ICU during the study observation period	3
Results for inter-rater reliability for inclusion decision	Describe the analysis results of inter-rater agreements/disagreements	3
Description of the characteristics of patients with ADE	Describe the studied population: age, gender, severity score at admission, reason for admission, origin of patients (home, hospital)	3
Number and classes of drugs suspected to be involved in the ADE responsible for ICU admission	The Anatomical Therapeutic Chemical classification should be used	3
Results for causality assessment	Provide all causality assessment results	3
Results for the preventability rate of ADE	Indicate the estimated preventability rate of ADE	3
Results for severity of ADE	Indicate how many patients died and how many required organ support	3
Results for ICU mortality rate of patients with and without ADE (separately)	Indicate and compare the ICU mortality rates of patients with and without ADE. Estimates of the hospital mortality rates for these patients would also be welcome	3
Length of stay in ICU of patients with and without ADE (separately)	Indicate and compare the lengths of stay in the ICU for patients with and without ADE	3
*Not mandatory*		
Results of inter-rater reliability for causality and preventability	Describe the analysis results of inter-rater agreements/disagreements	0.5
Research of medical causes that contributed to ADE (for preventable ADE)	For preventable ADE, investigations into the medical causes that contributed to ADE, such as prescription despite contraindication, dosage nonappropriate according to weight or specific pathologies (that is, renal impairment) may constitute valuable data to report	0.5
Number of drugs taken by patients with ADE prior to ICU admission	The total number of drugs taken by patients with ADE prior to ICU admission would be welcome, as well as drugs prescribed but not taken	0.25
Clinical features of ADE	Describe all clinical features of ADE	0.25
Comorbidities of patients with ADE	Describe comorbidities of patients with ADE	0.25
**Discussion**		
Limitations	Discuss limitations of the study, taking into account sources of potential bias	2
Interpretation	Interpret results and compare with previous studies	2

ADE, adverse drug events; SAPS II, Simplified Acute Physiology Score II; SOFA, Sequential Organ Failure Assessment.

#### Sensitivity analysis

To investigate heterogeneity in detail, analyses were conducted in which one or several included studies were removed. Such analyses included funnel and forest plots, and assessment of the *I*^2^ statistic.

## Results

### Study selection

**Figure 1:**
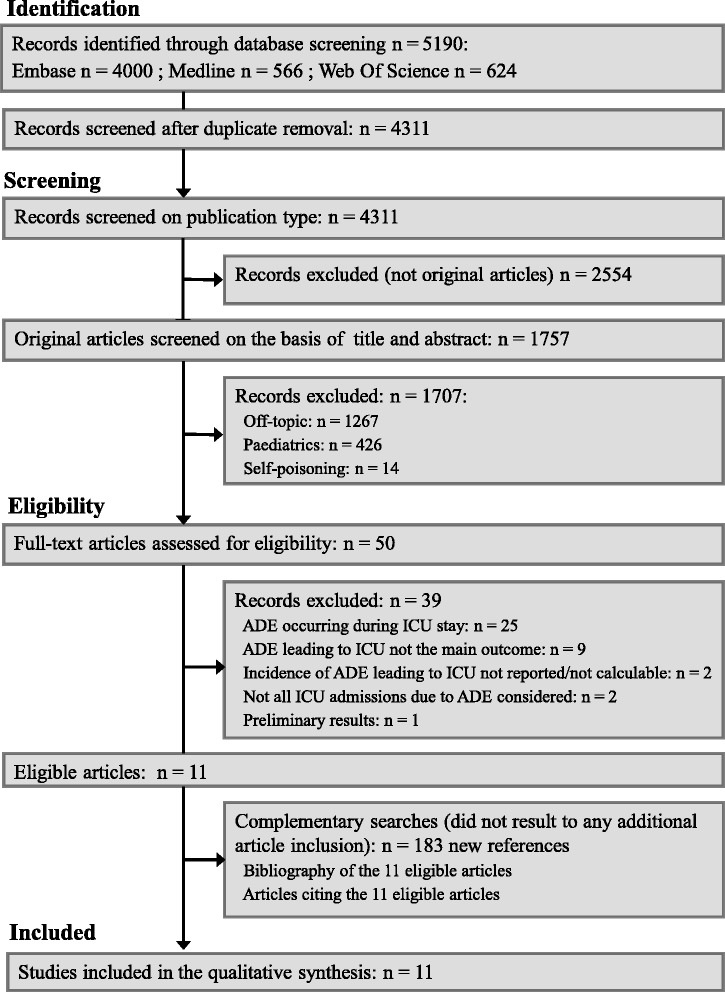
**Flow diagram of the literature search.** ADE, adverse drug events.

The initial queries yielded a total of 4,311 initial records (Figure 1). Among the 50 articles assessed for eligibility, full-text reading resulted in the exclusion of 39 (see Additional file 2 for exclusion reasons). The whole process resulted in the selection of 11 articles [10-20]. A particular article raised concerns for selection: a multicentre study gathering results from eight ICUs, one of which was a paediatric ICU. However, both reviewers selected this article because of the limited impact of the paediatric ICU in the global results of this multicentre study [15]. A disagreement requiring a third reviewer concerned only one article [12]. Neither the examination of the reference lists of the selected articles nor that of their citing lists (*n* =183 new references screened and assessed for eligibility) resulted in the retrieval of any additional article finally included in the review.

### Study characteristics

The 11 selected studies were published between 1986 and 2012 and were performed in different countries, types of hospital and ICUs (Table 3). Various features were reported, including incidence, cause, causality, risk factors, severity, preventability, length of stay and costs.

**Table 3:** **Design and aims of the selected studies**

**Study**	**Country**	**Study duration**	**Study design**	**Type of hospital**	**Type of ICU**	**Number of patients admitted during studied period**	**Aims of the study**
Trunet and colleagues, 1986 [10]	France	33 months (August 1978 to April 1981)	Prospective monocentre	Teaching hospital	Multidisciplinary ICU	1,651	Determine cause and effect relationship between drugs and adverse event, severity of DII, role of underlying disease and potential preventability of DII
IGICE, 1987 [11]	Italy	6 months	Prospective (data collection on a given day each week)	ND	27 general ICUs	4,537	Document aspects of ADR epidemiology in 27 general ICUs
Nelson and Talbert, 1996 [12]	USA	1 month (July to August 1993)	Prospective monocentre	Teaching hospital	Medical ICU, CCU and internal medicine service	127^a^	Describe the frequency and pattern of drug-related morbidity that results in hospital admission and the extent to which these admissions are avoidable
Darchy and colleagues, 1999 [13]	France	12 months (January to December 1994)	Retrospective monocentre	General hospital (500 beds)	Medico-surgical ICU (15 beds), CCU (6 beds)	623	Determine whether aging of the general population and medical advances have altered the incidence, causes and consequences of severe IDs, compared with Trunet and colleagues’ first study [21]
Hammerman and Kapeliovich, 2000 [14]	Israel	36 months (July 1994 to June 1997)	Prospective monocentre	Teaching hospital (900 beds)	CCU (9 beds)	2,559	Evaluate major cardiac iatrogenic disease as the cause of admission to the CCU
Lehmann and colleagues, 2005 [15]	USA	12 months (November 1998 to November 1999)	Prospective monocentre	Four teaching hospitals	Four surgical ICUs, three medical ICUs and one paediatric ICU	5,727	Identify the frequency and type of iatrogenic medical events requiring admission to the ICU. Assess the consequences of iatrogenic medical events for patients, and the incidence of disclosure of iatrogenic medical events to patients
Grenouillet-Delacre and colleagues, 2007 [16]	France	6 months (May to October 2003)	Prospective monocentre	Teaching hospital	Medical ICU	436	Assess the characteristics of life-threatening ADR in patients admitted to a medical ICU in order to identify associated risk factors that could facilitate early identification
Rivkin, 2007 [17]	USA	19 weeks (December 2004 to May 2005)	Prospective monocentre	Teaching hospital (1,076 beds)	Medical ICU (12 beds)	281	Determine frequency, severity and preventability of ADR leading to admission to a medical ICU
Schwake and colleagues, 2009 [18]	Germany	12 months (January to December 2003)	Prospective monocentre	Teaching hospital (1,685 beds)	Medical ICU (14 beds)	1,554	Determine the incidence of ICU admissions due to ADR and compare affected patients with patients admitted to the ICU for the treatment of deliberate self-poisoning using medical drugs
Mercier and colleagues, 2010 [19]	France	6 months (November 1999 to April 2000)	Prospective monocentre	Teaching hospital	Medical ICU (27 beds)	528	Determine the incidence, risk factors, severity and preventability of IEs as cause of ICU admission
Nazer and colleagues, 2013 [20]	Jordan	5 months (August to December 2010)	Prospective monocentre	Teaching cancer centre (170 beds)	Medico-surgical ICU (12 beds)	249	Describe the incidence, characteristics and cost of ADE that necessitate admission to the ICU in oncology patients

ADE, adverse drug events; ADR, adverse drug reactions; CCU, coronary care unit; DII, drug-induced illness; ID, iatrogenic disease; IE, iatrogenic event; IGICE, Italian Group on Intensive Care Evaluation; ND, not documented. ^a^Number of patients hospitalised in the medical ICU department.

### Risk of bias within studies

The scores of individual studies for the risk of bias ranged from 0.33 to 0.79 (Table 4), with six studies [10,13,16-18,20] having a score above 0.5 (one-half of the items completely reported) and five studies a score below 0.5 [11,12,14,15,19].

**Table 4:** **Risk of bias in individual studies**

	**Study**										
**Evaluated item**	**[** 10 **]**	**[** 11 **]**	**[** 12 **]**	**[** 13 **]**	**[** 14 **]**	**[** 15 **]**	**[** 16 **]**	**[** 17 **]**	**[** 18 **]**	**[** 19 **]**	**[** 20 **]**
Prospective study design?	1	1	1	0	1	1	1	1	1	1	1
Description of the type of studied ICU?	1	1	1	1	1	1	1	1	1	0	1
Complementary information on the setting environment?	0	0	0	1	0	0	0	1	1	0	0
Study size rationale?	0	0	0	0	0	0	0	0	0	0	0
Definition of ADE according to IOM?	0	0	0	0	0	0	0	0	0	0	1
Evaluation of inter-rater reliability for inclusion decision?	0	0	0	0	0	0	0	0	0	0	0
Description of evaluators’ training?	1	1	1	1	1	1	1	1	1	1	1
Description of patients’ screening?	1	1	1	1	1	1	1	1	1	1	1
Description of inclusion/exclusion criteria?	1	0	0	1	1	1	1	1	1	1	1
Description of collected data?	1	1	0	1	0	1	1	0	1	1	1
Description of the drug history collecting method?	0	0	0	0	0	0	1	0	0	0	0
Description of causality assessment method?	1	0	1	1	1	0	1	1	1	1	0
Description of preventability method/criteria?	0	0	1	1	1	1	1	1	0	1	1
Definition of ADE severity?	1	1	0	1	0	0	1	1	1	0	0
Description of study duration?	1	0	1	1	1	1	1	1	1	1	1
Results for incidence of ADE requiring ICU admission?	1	1	1	1	1	1	1	1	1	1	1
Results for inter-rater reliability for inclusion decision ?	0	0	0	0	0	0	0	0	0	0	0
Description of the characteristics of patients with ADE (age, gender, severity score at admission, reason of admission, origin of patients)?	0	1	0	0	0	0	1	0	0	0	0
Description of number and classes of drugs suspected to be involved in the ADE responsible for ICU admission?	1	0	0	1	1	0	1	0	1	1	1
Results for causality assessment?	1	0	0	1	0	0	1	1	0	0	0
Results for preventability rate?	0	0	0	1	1	0	1	1	0	1	1
Results for ADE severity?	1	1	0	1	0	0	1	1	1	0	1
Results for ICU mortality rate of patients with ADE?	1	1	0	1	0	0	1	1	1	0	1
Results for the length of stay for patients with and without ADE (separately)?	0	0	0	1	0	0	1	1	1	0	1
Proportion of items completely reported	0.58	0.42	0.33	0.71	0.46	0.38	0.79	0.67	0.63	0.46	0.63

ADE, adverse drug events; IOM, Institute of Medicine.

### Results of individual studies

#### Population characteristics

Inclusion and exclusion criteria were specified in nine studies and were heterogeneous across studies. The mean length of ICU stay for patients admitted to the ICU because of ADE was reported in five articles [13,16-18,20] and varied from 2.3 days [18] to 6.4 days [16]. In the study by Nazer and colleagues performed in Jordan, the average discounted cost (hospital bill for patient charge normalised in 2014 US dollars) for the management of an ICU admission related to ADE would amount to $10,388 with a median cost of $4,785 [20]. According to the payer’s perspective adopted in the study by Darchy and colleagues performed in France, the average discounted cost of an ICU admission related to ADE (including iatrogenic events related to surgery or procedures) would amount to $5,606 [13].

Patients specifically admitted to the ICU for ADE were compared with patients admitted to the ICU for reasons other than ADE in only three studies [13,16,20], and comparisons involved various criteria from one study to another (see Additional file 3).

#### Clarification of terminology and incidence of adverse drug events

Five studies reported the incidence of ADE as a cause for ICU admission, ranging from 0.37 to 22.9% [13-15,19,20] (Additional file 4). Five studies reported more specifically the incidence of ADR, ranging from 0.53 to 27.4% [11,12,16-18], and one study reported the combined incidence of ADE (5.9%), ADR (4.0%) and medication errors (1.9%) potentially responsible for ICU admission [10].

#### Characterisation of adverse drug events responsible for admissions to the ICU

The mortality rate ranged from 2% [18] to 28.1% [20]. The preventability rate varied between 17.5% [20] and 85.7% [17] (Table 5 and Additional file 3). Inter-rater agreement was only reported for the judgement of the preventability of iatrogenic medical events, and only in one study [15] – with a corresponding moderate inter-rater agreement (kappa test =0.5).

**Table 5:** **Description of the adverse drug events requiring admissions to the ICU**

**Study**	**Severity/type of scale and results, % (** ***n*** **)**	**Drugs involved** ^**a**^ **, % of patients** ***(n)***	**Preventability and categorisation if any**	**Preventability rate, % of patients (** ***n*** **)**
Trunet and colleagues, 1986 [10]	Fatal, 9.3% (9); life-threatening, 27.9% (27); severe, 35% (34); moderate, 27.8% (27)	Psychotropic drugs, 17.5% (17); anticoagulants, 13.4% (13); intravenous solutions, 12.4% (12); antibiotics, 11.4% (11); diuretics, 9.3% (9)	Not investigated	Not investigated
IGICE, 1987 [11]	Minor, 0%; major, 100%	30 drugs involved in ADR; single drug, 83.3% ADR (20); association of drugs, 16.7% ADR (4)	Not investigated	Not investigated
Nelson and Talbert, 1996 [12]	Not investigated	ND specifically for ICU patients: hypoglycaemic drugs, 15.8% (12)^b^; diuretics, 13.2% (10)^b^; anti-infectious drugs, 11.8% (9)^b^; cardiovascular drugs, 10.5% (8)^b^; psychotic drugs, 9.2% (7)^b^	Definitely avoidable – satisfied by one of the following criteria: patient did not take a prescribed drug, known allergy to the drug, contraindication between the drug and his disease/condition, the patient took a drug not prescribed or not indicated for a diagnosed disease	ND specifically for ICU patients: definitely avoidable, 49.3% (36)^b^
			Possibly avoidable: monitoring of the patient’s drug therapy not inadequate	Possibly avoidable, 9.6%^b^ (7)
			Not avoidable – no reasonable actions could have prevented it	Not avoidable, 37.0%^b^ (27)
			Unevaluable – information is insufficient to make a determination or is contradictory	Unevaluable, 4.1%^b^ (3)
Darchy and colleagues, 1999 [13]	Fatal, 14.6% (6); life-threatening, 12.2% (5); moderate, 73.2% (30)	55 drugs involved in ID; single drug, 22 IDs; association of drugs, 19 IDs; diuretics, 17.1% (7); oral anticoagulants, 14.6% (6); nonsteroidal anti-inflammatory, 14.6% (6); antibiotics, 14.6% (6); anaesthesia, 12.2% (5)	Event that should not occur if management is the best that medical science can provide	73.1% (30)
Hammerman and Kapeliovich, 2000 [14]	Not investigated	234 drugs involved in major IE; single drug, one major IE; association of drugs, 63 major IEs; nitrates, 76.6% (49); diuretics, 70.3% (45); beta-blockers, 68.8% (44); ACE inhibitors, 45.3% (29); calcium antagonists, 43.8% (28)	Event that could have been avoided if the prescription of therapy had respected the art of medical practice	64.1% (41)
Lehmann and colleagues, 2005 [15]	Not investigated	Narcotic analgesics, 42.8% (9); sedative hypnotics, 23.8% (5)	Event avoidable using any means currently available, unless those means where not considered standard of care [22]	ND specifically for ICU patients: preventable, 34.4% (22); not preventable, 14.1% (9); not assessable, 51.6% (33)
Grenouillet-Delacre and colleagues, 2007 [16]	According to World Health Organization [23]: life-threatening, 94% (124); potentially life-threatening, 6.0% (8); among these events, 15.9% (21) contributed to death	132 drugs involved; psychotropic drugs, 22.5% (25); immunosuppressive drugs, 21.6% (24); anticoagulant drugs, 13.5% (15); anti-infectious drugs, 12.6% (14); antihypertensive drugs, 12.6% (14)	Definitely preventable – all conditions for avoidance of its occurrence were fulfilled	Definitely preventable, 10.6% (14)
			Potentially preventable – not all conditions were met to avoid its occurrence	Potentially preventable, 37.1% (49)
			Not preventable – treatment procedure consistent with current knowledge of good medical practice	Not preventable, 21.2% (28)
				Not assessable (lack of data), 31.1% (41)
Rivkin, 2007 [17]	Fatal, 19% (4); severe, 66.7% (14); moderate, 14.3% (3)	39 drugs involved; single drug, 43% ADR (9); associations of drugs, 57% ADR (12)	Medication use was inappropriate and contrary to standard clinical practice [24]	85.7% (18)
Schwake and colleagues, 2009 [18]	Life-threatening, 37.4% (37); potentially life-threatening, 62.6% (62); Among these events 2% (2) were fatal	anticoagulants, 62.6% (62); analgesics, 25.2% (25); diuretics, 16.2% (16); antihypertensives, 5% (5); antidepressants, 5% (5)	Not investigated	Not investigated
Mercier and colleagues, 2010 [19]	Not investigated	54 drugs involved; chemotherapy, immunosuppressant drugs, 27.8% (15); psychotropic drugs, 14.8% (8); cardiovascular drugs, 14.8% (8); anaesthesia, analgesic drugs, 11.1% (6); oral anticoagulants, 9.3% (5)	Preventable ADR: drug not used according to the summary of product characteristic	64% (32)
Nazer and colleagues, 2012 [20]	Fatal, 28.1% (16); life-threatening, 17.6% (10); significant, 54.3% (31)	Antineoplastic drugs: 64.9% (37); analgesics: 15.8% (9); anticoagulants: 7% (4); others: 12.3% (7)	ADE met at least one of the following criteria: inappropriate drug or unnecessary for the patient’s condition, drug dose; route or frequency inappropriate for the patient’s age, weight or disease state; required supportive/preventive therapies not prescribed; required therapeutic drug monitoring or laboratory tests not performed; history of allergy; resulted from a well-established drug interaction	17.5% (10)

ACE, angiotensin-converting enzyme; ADE, adverse drug events; ADR, adverse drug reaction; ID, iatrogenic disease; IE, iatrogenic event; IGICE, Italian Group on Intensive Care Evaluation; ND, not documented. ^a^The five most frequent classes of drugs (when mentioned) are shown. ^b^Pooled result of the three studied departments.

The leading causes of ADE were reported briefly. Trunet and colleagues underlined that ADE were due to 12 drug–drug interactions and 31 medication errors (28 overdosages and three cases of drug prescription in spite of contraindications) [10]. Darchy and colleagues described the causes of the 30 ADE: inadequate follow-up of therapy in 46.7% (14 cases), error in dose in 26.7% (eight cases), inappropriate drug in 20% (six cases) and failure to use prophylactic treatment in 6.6% (two cases) [13]. Lehmann and colleagues categorised ADE into dosage error in 43% (nine cases), idiosyncratic reaction in 33% (seven cases), frequency error in 10% (two cases), unclassified error in 10% (two cases) and wrong drug to patient in 5% (one case) [15]. Grenouillet-Delacre and colleagues mentioned that 87 of the 132 ADRs (66%) were caused by interaction between drugs [16]. Finally, anaphylaxis or improper drug use were mentioned in the study by the Italian Group on Intensive Care Evaluation [11].

The most frequent drugs involved in ADE in the 11 studies were cardiovascular, anticoagulant and psychotropic drugs. Most ADE were preventable but the processes that had led to the corresponding drug misuse were poorly detailed.

#### Causality results

As shown in Table 6, the data required for assessing causality differ from one method to another. Various causality assessments were used, and results on causal assessments of ICU admissions related to ADE were mentioned in only four articles [10,13,16,17].

**Table 6:** **Causality results**

**Required data or study**	**Causality assessment methods**					
	**Kramer and colleagues** **[** 25 **]**	**WHO** **[** 26 **]**	**Naranjo and colleagues** **[** 27 **]**	**Karch–Lasagna** **[** 28 **]**	**Hallas and colleagues** **[** 29 **]**	**Begaud and colleagues** **[** 30 **]**
Required data for causality assessment	L, Chron, D, R, LT, AEC	L, Chron, D, R, AEC, PP	L, Chron, D, R, Pl, DM, LT, AEC, Atcd, OE	L, Chron, D, R, AEC	Chron, D, R, LT, AET, Atcd	L, Chron, D, R, LT, AEC, PP
Trunet and colleagues, 1986 [10]	de, 29.7% (30); pr, 45.6% (46); po, 20.8% (21); un, 3.9% (4)					
IGICE, 1987 [11]^a^						
Nelson and Talbert, 1996 [12]			pr, 59.6% (31)^b^; po, 40.4% (21)^b^		de, 15.3% (8)^b^; pr, 40.4% (21)^b^; po, 25% (13)^b^; un, 19.3% (10)^b^	
Darchy and colleagues, 1999 [13]				de, 34.1% (14); pr, 34.1% (14); po, 31.8% (13)		
Hammerman and Kapeliovich, 2000 [14]				ND		
Lehmann and colleagues, 2005 [15]				ND		
Grenouillet-Delacre and colleagues, 2007 [16]			ND			Very likely, 8.3% (11); likely, 51.5% (68); possible, 40.2% (53)
Rivkin, 2007 [17]			de, 4.8% (1); pr, 80.9% (17); po, 14.3% (3)			
Schwake and colleagues, 2009 [18]		ND				
Mercier and colleagues, 2010 [19]				ND		
Nazer and colleagues, 2013 [20]^a^						

IGICE, Italian Group on Intensive Care Evaluation; ND, not documented; WHO, World Health Organization. Results of causality assessment methods: de, definite; po, possible; pr, probable; un, unlikely. Required data used for assessing causality in each method: AEC, alternative etiologic candidates (other than drugs); Atcd, antecedent of similar event to the same drug; Chron, chronology; D, clinical outcome after dechallenge; DM, clinical outcome after dose modification; L, description in the literature; LT, results of therapeutic drug monitoring or laboratory test; OE, adverse event confirmed by objective evidence; Pl, clinical outcome after placebo administration; PP, explanation by pharmacologic properties; R, clinical outcome after rechallenge. ^a^This study did not report causality assessment. ^b^Pooled results for the three departments.

### Risk of bias across studies

**Figure 2:**
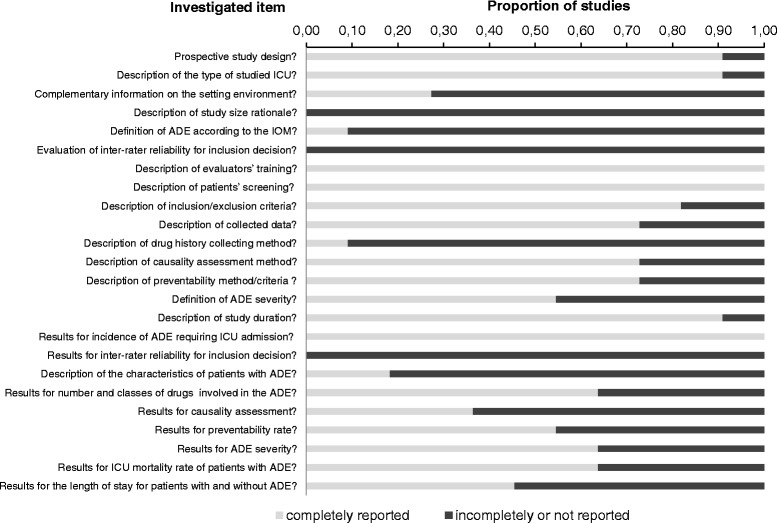
**Risk of bias across studies.** ADE, adverse drug events; IOM, Institute of Medicine.

Considering the 24 items retained for assessing the risk of bias in/across studies, nine (37%) items were not reported or were incompletely reported in at least one-half of the studies (Figure 2), with five items related to methodology (out of 15) and four items related to results (out of nine).

### Synthesis of results

**Figure 3:**
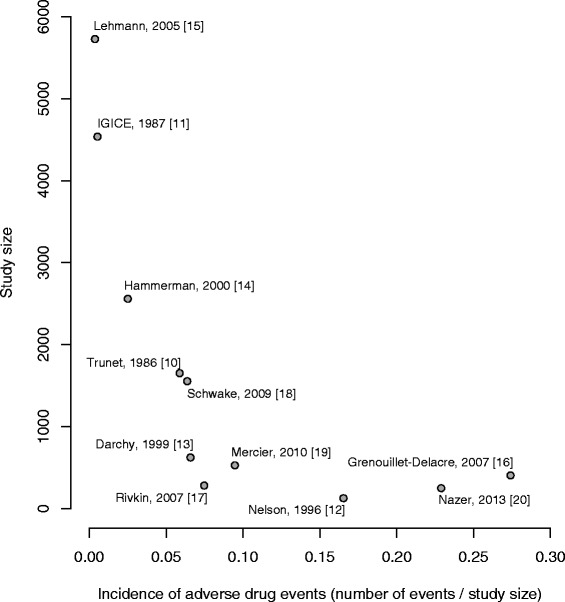
**Funnel plot of the incidence of admissions to the ICU required due to adverse drug events** **[** 10-20 **]** **.** IGICE, Italian Group on Intensive Care Evaluation.

**Figure 4:**
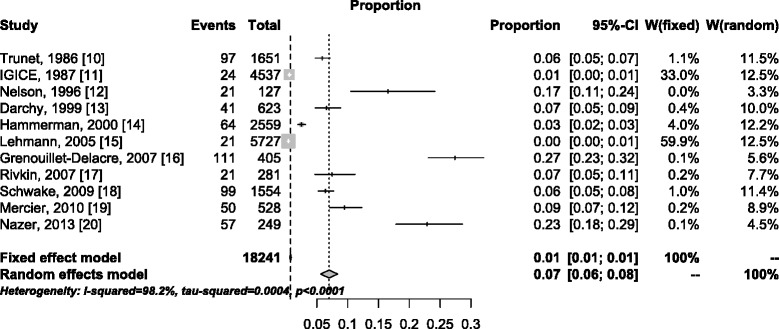
**Forest plot of the incidence of admissions to the ICU required due to adverse drug events** **[** 10 **-** 20 **]** **.** CI, confidence interval; IGICE, Italian Group on Intensive Care Evaluation.

The asymmetric shape of the funnel plot shown in Figure 3 indicates that large studies tend to report low estimates of the IDRIA. A forest plot adopting a random effects model resulted in a final estimate of the IDRIA at 7% (6%; 8%), with a total sample size of 18,241 patients (Figure 4). However, because of the large heterogeneity of the studies (*I*^2^ statistic >98%), the above final estimate should not be considered a summary measure.

### Additional analyses

#### Quality assessment

**Figure 5:**
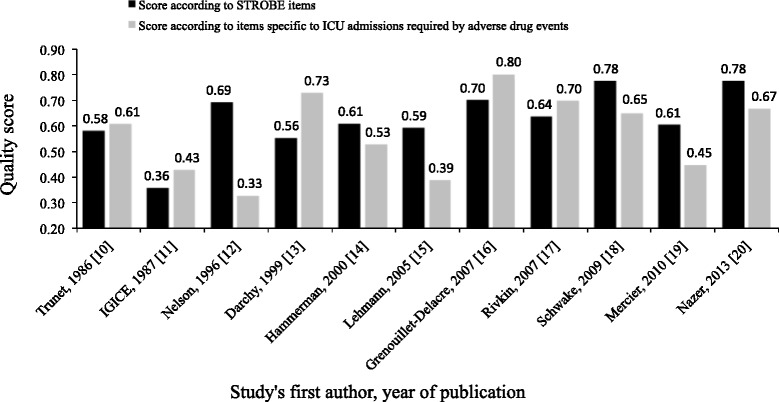
**Quality assessment of the studies according to STROBE items** **[** 7 **]** **and according to specific items of adverse drug events responsible for ICU admission** **[** 10 **-** 20 **]** **.** IGICE, Italian Group on Intensive Care Evaluation; STROBE, Strengthening the Reporting of Observational Studies in Epidemiology.

The quality scores of the included studies varied from 0.36 [11] to 0.78 [18,20], with a median (interquartile range) value of 0.61 (0.59; 0.70), when assessment was based on STROBE items (Figure 5). The scores varied from 0.33 [12] to 0.80 [16] with a median (interquartile) value of 0.61 (0.44; 0.69) when quality assessment was based on the checklist that we devised for considering specific items on ADE resulting in ICU admission (Table 2). The differences between the two scores varied highly from one study to another (Figure 5).

#### Sensitivity analysis

A first analysis was conducted with removal of the study by Lehmann and colleagues [15] (highest sample size and extremely low estimate of the IDRIA). A second analysis was conducted with the removal of the studies by Lehmann and colleagues and by the Italian Group on Intensive Care Evaluation (the two studies with the highest sample sizes and the lowest estimates of the IDRIA) [11,15]. A third analysis was conducted considering the six studies with the lowest risk of bias [10,13,16-18,20] (see Table 4). The corresponding forest plots of these three analyses (data not shown) still indicated a large heterogeneity across the considered studies, and the *I*^2^ statistic always remained above 96%, confirming such heterogeneity.

## Discussion

Our review of published reports regarding ICU admissions related to ADE identified 11 original studies. The IDRIA reported ranged from 0.37% [15] to 27.4% [16].

Vlayen and colleagues proposed a review on the incidence and preventability of adverse events that required intensive care (re)admission [31]. The adverse events considered by Vlayen and colleagues not only included ADE but also surgical procedures and medical devices, the population considered was adult and paediatric patients originating from hospital wards only, and the incidence of events was reported according to the total number of stays in the studied hospital ward(s). The perspective of our review was different: the events considered were strictly ADE as a cause of ICU admission, the patient population was adult patients (whatever the origin of the patient), and the incidence was reported according to the total number of admissions in ICU. These important perspective differences probably explain the fact that nine of the 11 articles retained in our study were not included in Vlayen and colleagues’ study selection [31].

Our study outlines several methodological aspects that contribute to the observed wide range of the reported IDRIA, such as study setting (types of patients and ICU under study) and the type of drug-related events considered. The definition of ADE that was proposed by the Institute of Medicine and recommended by Nebeker and colleagues [4] should be adopted as it encompasses all aspects of ADE.

The methods used for assessing preventability of ADE also varied across studies, and either implicit or explicit criteria were used. Excepted for one study gathering a large proportion of febrile neutropenia, which is not preventable [20], the preventability rate in the seven other studies was high (lowest estimate 47.7% [16]). However, a recent systematic review on the methods for assessing the preventability of ADE concludes that there is limited evidence for the validity of the identified instruments, and instrument reliability varied significantly [32]. Moreover, the single study that assessed inter-rater agreement for determining preventability resulted in a moderate agreement [15]. All in all, a reliable and reproducible assessment of preventability remains a challenge at the present time.

Another source of heterogeneity was related to the causality assessment method. Kane-Gill and colleagues compared causality assessment methods (Naranjo, Kramer and Jones) in the ICU [33]. Agreement between the methods depended on whether events were judged retrospectively or were issued from a surveillance monitoring system. At the present time, the degree of agreement/disagreement between methods remains unclear. Most of all, existing tools widely used across different countries have not been customised to assess causality in the ICU. For example, the requirement of a procedure of rechallenge and dechallenge is a key element for assessing the certainty of the causality in the Naranjo, the Karch–Lasagna and the World Health Organization assessment methods. While one can understand the value of such an element with regard to causality strictly speaking, rechallenge is not possible most of the time in patients admitted to the ICU because of the seriousness of the drug-related events and poor patient health status. Moreover, the highest score for causality with these methods implies an improvement of the clinical features after dechallenge whereas recovery after dechallenge may be not achieved in ICU patients (irreversible organ failure, long-lasting effects of ADE). The events and patients involved in intensive care are therefore not well adapted to the above causality scales and, all in all, the design of a specific algorithm tailored to ICU cases would be welcome. Nevertheless, at the present time, the French official method by Begaud and colleagues, in which rechallenge or dechallenge is not a mandatory item for high causality scores, appears the best available tool [30].

Unsurprisingly, the most frequent drugs involved in ADE in the 11 studies are the same drugs that cause admission to emergency departments [34-36]. However, in order to better assess the dangerousness of these drugs, the frequencies of these ADE should be contrasted with the prescribing frequencies of the corresponding drugs. Exploring these relationships constitutes an important issue that deserves investigation. These investigations would facilitate the identification of specific patterns related to preventable ADE requiring ICU admissions. In that regard, the use of effective identification and reporting systems based on a consistent terminology should contribute to the design of preventive measures for avoiding such events.

In our review, quality assessment of the 11 articles not only included global STROBE recommendations but also items specific to the domain of critical care. The quality of reporting assessed according to STROBE items and according to our proposed items (see Table 2) resulted in identical median scores of 0.61 (see Figure 5), indicating that the quality of reporting for future research is substantially improvable. In addition, the within-study difference between the two above quality scores (Figure 5) indicates that the quality of reporting with regards to the global form (quality according to STROBE guidelines) may substantially differ from the value of the information reported according to a perspective focusing on the report of ADE in intensive care patients (quality of items specific to the ICU).

In this regard, our corresponding specific items might also be considered as a potential future checklist: experts in critical care could use this list and the associated scores as an initial proposal for developing international guidelines aimed at improving the quality of future research on ADE-related ICU admissions. The use of this checklist should also decrease the risk of bias within and across studies.

This review has some limitations. The fact that only 11 studies were published during the last three decades may be related to two major elements. First, investigations into ADE in the intensive care setting require time-consuming resources and complex data. In that regard, the expanding availability of healthcare systems based on electronic medical records should facilitate future research on large cohorts of patients. Second, publication bias should be considered: one might hypothesise that studies reporting a high frequency of ADE requiring ICU admission are less likely to be published than those associated with a low estimate. The retrieved studies show a wide range for the IDRIA, however, and publication bias may therefore be limited. In addition, we only selected articles written in English, because we did not have the resources available to translate potential articles written in various other languages. Moreover, when considering a given study, only the quality of reporting was assessed. The major and important limitation of the review concerns the heterogeneity observed across studies for the definitions of events (ADE, ADR) and for the methods used for causality, preventability and severity assessment. Forest plot analyses of the IDRIA reflect such heterogeneity, which remained very high in the sensitivity analyses excluding some studies. Similarly, the asymmetric shape of the funnel plot also raises concerns. Heterogeneity and biases therefore finally constitute the main issue of the present review, in contrast to other reviews for which the final estimate issued from the meta-analysis is the main result.

## Conclusion

Few studies dealt with ADE as a cause of admission to the ICU during the last three decades. The IDRIA reported varied within a large range, from 0.37 to 27.4%. Severity evaluation and the mortality rate are characteristics of major importance in ICU patient populations and these features should be explored and analysed in future studies. To encompass all aspects of ADE, compliance issues as well as lack of access to care should be investigated. Finally, methodological aspects should be enhanced in order to improve the quality of reports and limit risks of bias. In this regard, we propose a checklist (Table 2) specifically tailored to the topic of ADE requiring ICU admission that could be used as a helpful guide for future studies.

## Additional files

Additional file 1:
**List of the search strategies.**
Additional file 2: Table AF1. Assessment for eligibility of full-text articles: the 39 excluded articles. Additional file 3: Table AF2. Population characteristics. Additional file 4: Table AF3. Clarification of event terminology and incidence of event-related admissions in the ICU.

## Abbreviations

ADE: Adverse drug events
ADR: Adverse drug reaction
IDRIA: Incidence of drug-related ICU admissions
STROBE: Strengthening the Reporting of Observational Studies in Epidemiology
