# Supplementary material for: A systematic review of adult admissions to ICUs related to adverse drug events
Source: Crit Care. 2014 Nov 25;18(6):643. doi: 10.1186/s13054-014-0643-5 (PMC4422001; doi:10.1186/s13054-014-0643-5)
Supplement: Additional file 1: — List of the search strategies. [file 13054_2014_643_MOESM1_ESM.docx]

**Additional file 1. Search Strategies**

PubMed:

#1 Search "adverse drug event" OR "adverse drug events"

#2 Search "adverse drug reaction" OR "adverse drug reactions"

#3 Search iatrogenic

#4 Search "side effect" OR "side effects"

#5 Search "intensive care"[TW] OR icu

#6 Search admission*

#7 Search admitted

#8 Search 1982/01/01:2014/07/02[DP]

#9 Search English[LA]

#10 Search #1 OR #2 OR #3 OR #4

#11 Search #6 OR #7

#12 Search #5 AND #10 AND #11 AND #8 AND #9

#13 Search "Iatrogenic Disease"[Mesh]

#14 Search "Drug-Related Side Effects and Adverse Reactions"[Mesh]

#15 Search "Intensive Care"[Mesh:NoExp]

#16 Search "Intensive Care Units"[Mesh]

#17 Search #13 OR #14

#18 Search #15 OR #16

#19 Search (#17 AND #18 AND #11 AND #8 AND #9)

#20 Search (#12 OR #19)

Embase:

#1 'intensive care unit':de,ab,ti AND [embase]/lim

#2 'intensive care units':de,ab,ti AND [embase]/lim

#3 icu:de,ab,ti AND [embase]/lim

#4 'intensive care':de,ab,ti AND [embase]/lim

#5 iatrogenic AND [embase]/lim

#6 side NEXT/1 effect* AND [embase]/lim

#7 adverse NEXT/1 drug AND [embase]/lim

#8 drug NEXT/1 reaction* AND [embase]/lim

#9 drug NEXT/1 event* AND [embase]/lim

#10 adverse NEXT/1 event* AND [embase]/lim

#11 drug OR drugs AND [embase]/lim

#12 #7 AND #8

#13 #7 AND #9

#14 #10 AND #11

#15 admission* AND [embase]/lim

#16 admitted AND [embase]/lim

#17 #1 OR #2 OR #3 OR #4

#18 #5 OR #6 OR #12 OR #13 OR #14

#19 #15 OR #16

#20 #17 AND #18 AND #19

#21 #20 AND [english]/lim AND [embase]/lim AND [1982-2014]/py

#22 'intensive care unit'/exp AND [embase]/lim

#23 'intensive care'/exp AND [embase]/lim

#24 'adverse drug reaction'/exp AND [embase]/lim

#25 'side effect'/exp AND [embase]/lim

#26 'iatrogenic disease'/de AND [embase]/lim

#27 #22 OR #23

#28 #24 OR #25 OR #26

#29 #19 AND #27 AND #28

#30 #29 AND [english]/lim AND [embase]/lim AND [1982-2014]/py

#31 #21 OR #30

Web of Science:

#1 TOPIC: (intensive care) Indexes=SCI-EXPANDED, SSCI, A&HCI, CPCI-S, CPCI-SSH Timespan=All years

#2 TOPIC: (intensive care unit*) Indexes=SCI-EXPANDED, SSCI, A&HCI, CPCI-S, CPCI-SSH Timespan=All years

#3 TOPIC: (icu) Indexes=SCI-EXPANDED, SSCI, A&HCI, CPCI-S, CPCI-SSH Timespan=All years

#4 TOPIC: (iatrogenic) Indexes=SCI-EXPANDED, SSCI, A&HCI, CPCI-S, CPCI-SSH Timespan=All years

#5 TOPIC: (side effect*) Indexes=SCI-EXPANDED, SSCI, A&HCI, CPCI-S, CPCI-SSH Timespan=All years

#6 TOPIC: (adverse drug reaction*) Indexes=SCI-EXPANDED, SSCI, A&HCI, CPCI-S, CPCI-SSH Timespan=All years

#7 TOPIC: (adverse drug event*) Indexes=SCI-EXPANDED, SSCI, A&HCI, CPCI-S, CPCI-SSH Timespan=All years

#8 TOPIC: (admission*) Indexes=SCI-EXPANDED, SSCI, A&HCI, CPCI-S, CPCI-SSH Timespan=All years

#9 TOPIC: (admitted) Indexes=SCI-EXPANDED, SSCI, A&HCI, CPCI-S, CPCI-SSH Timespan=All years

#10 #3 OR #2 OR #1 Indexes=SCI-EXPANDED, SSCI, A&HCI, CPCI-S, CPCI-SSH Timespan=All years

#11 #7 OR #6 OR #5 OR #4 Indexes=SCI-EXPANDED, SSCI, A&HCI, CPCI-S, CPCI-SSH Timespan=All years

#12 #9 OR #8 Indexes=SCI-EXPANDED, SSCI, A&HCI, CPCI-S, CPCI-SSH Timespan=All years

#13 #12 AND #11 AND #10 Indexes=SCI-EXPANDED, SSCI, A&HCI, CPCI-S, CPCI-SSH Timespan=All years

#14 (#13) AND LANGUAGE: (English) Indexes=SCI-EXPANDED, SSCI, A&HCI, CPCI-S, CPCI-SSH Timespan=1982-2014
