# Supplementary material for: A systematic review of adult admissions to ICUs related to adverse drug events
Source: Crit Care. 2014 Nov 25;18(6):643. doi: 10.1186/s13054-014-0643-5 (PMC4422001; doi:10.1186/s13054-014-0643-5)
Supplement: Additional file 2: Table AF1. — Assessment for eligibility of full-text articles: the 39 excluded articles. [file 13054_2014_643_MOESM2_ESM.docx]

**Additional File 2**

**Table AF1. Assessment for eligibility on full-text articles: the 39 excluded articles.**

| Reason of exclusion |  | Citations |
| --- | --- | --- |
| ADE occurring during ICU stay |  | [[1-25](#_ENREF_1)] |
| ADE leading to ICU: not the main outcome |  | [[26-34](#_ENREF_26)] |
| Incidence of ADE leading to ICU not reported/not calculable |  | [[35](#_ENREF_35), [36](#_ENREF_36)] |
| Not all ICU admissions due to ADE considered |  | [[37](#_ENREF_37), [38](#_ENREF_38)] |
| Preliminary results |  | [[39](#_ENREF_39)] |

**References AF1.**

1. Aljadhey H, Mahmoud MA, Mayet A, Alshaikh M, Ahmed Y, Murray MD, Bates DW: **Incidence of adverse drug events in an academic hospital: A prospective cohort study**. *International Journal for Quality in Health Care* 2013, **25**(6):648-655.

2. Alsbou M: **Incidence of adverse drug reactions in alkarak hospital: A pilot study**. *Jordan Medical Journal* 2010, **44**(4):442-446.

3. Al-Tajir GK, Kelly WN: **Epidemiology, comparative methods of detection, and preventability of adverse drug events**. *Ann Pharmacother* 2005, **39**(7-8):1169-1174.

4. Aranaz-Andres JM, Aibar-Remon C, Limon-Ramirez R, Amarilla A, Restrepo FR, Urroz O, Sarabia O, Garcia-Corcuera LV, Terol-Garcia E, Agra-Varela Y *et al*: **Prevalence of adverse events in the hospitals of five Latin American countries: Results of the 'Iberoamerican study of adverse events' (IBEAS)**. *BMJ Quality and Safety* 2011, **20**(12):1043-1051.

5. Ayub MN, da Silva D, Martinbiancho JK, Dal-Pizzol TS: **Adverse drug reactions in patients hospitalized in the intensive care unit of a university hospital in southern Brazil**. *Latin American Journal of Pharmacy* 2010, **29**(5):688-693.

6. Bates DW, Cullen DJ, Laird N, Petersen LA, Small SD, Servi D, Laffel G, Sweitzer BJ, Shea BF, Hallisey R *et al*: **Incidence of adverse drug events and potential adverse drug events: Implications for prevention**. *Journal of the American Medical Association* 1995, **274**(1):29-34.

7. Cullen DJ, Sweitzer BJ, Bates DW, Burdick E, Edmondson A, Leape LL: **Preventable adverse drug events in hospitalized patients: a comparative study of intensive care and general care units**. *Crit Care Med* 1997, **25**(8):1289-1297.

8. Devi P, Kamath DY, Anthony N, Santosh S, Dias B: **Patterns, predictors and preventability of adverse drug reactions in the coronary care unit of a tertiary care hospital**. *Eur J Clin Pharmacol* 2012, **68**(4):427-433.

9. Forster AJ, Worthington JR, Hawken S, Bourke M, Rubens F, Shojania K, van Walraven C: **Using prospective clinical surveillance to identify adverse events in hospital**. *Bmj Quality & Safety* 2011, **20**(9):756-763.

10. Giraud T, Dhainaut JF, Vaxelaire JF, Joseph T, Journois D, Bleichner G, Sollet JP, Chevret S, Monsallier JF: **Iatrogenic complications in adult intensive care units: A prospective two- center study**. *Crit Care Med* 1993, **21**(1):40-51.

11. Graf J, von den Driesch A, Koch KC, Janssens U: **Identification and characterization of errors and incidents in a medical intensive care unit**. *Acta Anaesthesiologica Scandinavica* 2005, **49**(7):930-939.

12. Hart GK, Baldwin I, Gutteridge G, Ford J: **Adverse incident reporting in intensive care**. *Anaesth Intensive Care* 1994, **22**(5):556-561.

13. Jennane N, Madani N, Oulderrkhis R, Abidi K, Khoudri I, Belayachi J, Dendane T, Zeggwagh A, Abouqal R: **Incidence of medication errors in a Moroccan medical intensive care unit**. *International Archives of Medicine* 2011, **4**(1).

14. Kathiria JM, Sattigeri BM, Desai PM, Patel SP: **A study of adverse drug reactions in patients admitted to intensive care unit of a tertiary care teaching rural hospital**. *International Journal of Pharmacy and Pharmaceutical Sciences* 2013, **5**(1):160-163.

15. Morimoto T, Sakuma M, Matsui K, Kuramoto N, Toshiro J, Murakami J, Fukui T, Saito M, Hiraide A, Bates DW: **Incidence of adverse drug events and medication errors in Japan: the JADE study**. *J Gen Intern Med* 2011, **26**(2):148-153.

16. Nazer LH, Hawari F, Al-Najjar T: **Adverse drug events in critically ill patients with cancer: Incidence, characteristics, and outcomes**. *Journal of Pharmacy Practice* 2014, **27**(2):208-213.

17. Nebeker JR, Hoffman JM, Weir CR, Bennett CL, Hurdle JF: **High rates of adverse drug events in a highly computerized hospital**. *Arch Intern Med* 2005, **165**(10):1111-1116.

18. Pagnamenta A, Rabito G, Arosio A, Perren A, Malacrida R, Barazzoni F, Domenighetti G: **Adverse event reporting in adult intensive care units and the impact of a multifaceted intervention on drug-related adverse events**. *Annals of Intensive Care* 2012, **2**(1).

19. Park S, In Y, Suh GY, Sohn K, Kim E: **Evaluation of adverse drug reactions in medical intensive care units**. *Eur J Clin Pharmacol* 2013, **69**(1):119-131.

20. Rahim SA, Mody A, Pickering J, Devereaux PJ, Yusuf S: **Iatrogenic adverse events in the coronary care unit**. *Circ Cardiovasc Qual Outcomes* 2009, **2**(5):437-442.

21. Rothschild JM, Landrigan CP, Cronin JW, Kaushal R, Lockley SW, Burdick E, Stone PH, Lilly CM, Katz JT, Czeisler CA *et al*: **The Critical Care Safety Study: The incidence and nature of adverse events and serious medical errors in intensive care**. *Crit Care Med* 2005, **33**(8):1694-1700.

22. Seynaeve S, Verbrugghe W, Claes B, Vandenplas D, Reyntiens D, Jorens PG: **Adverse drug events in intensive care units: a cross-sectional study of prevalence and risk factors**. *Am J Crit Care* 2011, **20**(6):e131-140.

23. Smith KM, Jeske CS, Young B, Hatton J: **Prevalence and characteristics of adverse drug reactions in neurosurgical intensive care patients**. *Neurosurgery* 2006, **58**(3).

24. Vargas E, Simon J, Martin JC, Puerro M, Gonzalez-Callejo MA, Jaime M, Gomez-Mayoral B, Duque F, Gomez-Delgado A, Moreno A: **Effect of adverse drug reactions on length of stay in intensive care units**. *Clinical Drug Investigation* 1998, **15**(4):353-360.

25. Vargas E, Terleira A, Hernando F, Perez E, Cordon C, Moreno A, Portoles A: **Effect of adverse drug reactions on length of stay in surgical intensive care units**. *Crit Care Med* 2003, **31**(3):694-698.

26. Anthes AM, Harinstein LM, Smithburger PL, Seybert AL, Kane-Gill SL: **Improving adverse drug event detection in critically ill patients through screening intensive care unit transfer summaries**. *Pharmacoepidemiology and Drug Safety* 2013, **22**(5):510-516.

27. Chan ALF, Lee HY, Ho CH, Cham TM, Lin SJ: **Cost evaluation of adverse drug reactions in hospitalized patients in Taiwan: A prospective, descriptive, observational study**. *Current Therapeutic Research - Clinical and Experimental* 2008, **69**(2):118-129.

28. Dartnell JGA, Anderson RP, Chohan V, Galbraith KJ, Lyon MEH, Nestor PJ, Moulds RFW: **Hospitalisation for adverse events related to drug therapy: Incidence, avoidability and costs**. *Medical Journal of Australia* 1996, **164**(11):659-662.

29. Doshi MS, Patel PP, Shah SP, Dikshit RK: **Intensive monitoring of adverse drug reactions in hospitalized patients of two medical units at a tertiary care teaching hospital**. *Journal of Pharmacology and Pharmacotherapeutics* 2012, **3**(4):308-313.

30. Johnston PE, France DJ, Byrne DW, Murff HJ, Lee B, Stiles RA, Speroff T: **Assessment of adverse drug events among patients in a tertiary care medical center**. *American Journal of Health-System Pharmacy* 2006, **63**(22):2218-2227.

31. McDonnell PJ, Jacobs MR: **Hospital admissions resulting from preventable adverse drug reactions**. *Ann Pharmacother* 2002, **36**(9):1331-1336.

32. Perez Menendez-Conde C, Bermejo Vicedo T, Delgado Silveira E, Carretero Accame E: **Adverse drug reactions which provoke hospital admission**. *Farm Hosp* 2011, **35**(5):236-243.

33. Raut A, Diwan A, Patel C, Patel P, Pawar A: **Incidence, severity and financial burden associated with adverse drug reactions in medicine inpatients**. *Asian Journal of Pharmaceutical and Clinical Research* 2011, **4**(SUPPL. 2):107-111.

34. Smith CC, Bennett PM, Pearce HM, Harrison PI, Reynolds DJM, Aronson JK, Grahame-Smith DG: **Adverse drug reactions in a hospital general medical unit meriting notification to the Committee on Safety of Medicines**. *British Journal of Clinical Pharmacology* 1996, **42**(4):423-429.

35. Garry DA, McKechnie SR, Culliford DJ, Ezra M, Garry PS, Loveland RC, Sharma VV, Walden AP, Keating LM: **A prospective multicentre observational study of adverse iatrogenic events and substandard care preceding intensive care unit admission (PREVENT)**. *Anaesthesia* 2014, **69**(2):137-142.

36. Joshua L, Devi P, Guido S: **Adverse drug reactions in medical intensive care unit of a tertiary care hospital**. *Pharmacoepidemiology and Drug Safety* 2009, **18**(7):639-645.

37. Arranto CA, Mueller C, Hunziker PR, Marsch SC, Eriksson U: **Adverse cardiac events in ICU patients with presumptive antidepressant overdose**. *Swiss Med Wkly* 2003, **133**(35-36):479-483.

38. Bapoje SR, Gaudiani JL, Narayanan V, Albert RK: **Unplanned transfers to a medical intensive care unit: Causes and relationship to preventable errors in care**. *Journal of Hospital Medicine* 2011, **6**(2):68-72.

39. Trunet P, Le Gall JR, Lhoste F, Rapin M: **Admissions to intensive care units for iatrogenic diseases**. *Legal Medicine* 1982, **Vol. 1982**:73-84.
