# Supplementary material for: A systematic review of adult admissions to ICUs related to adverse drug events
Source: Crit Care. 2014 Nov 25;18(6):643. doi: 10.1186/s13054-014-0643-5 (PMC4422001; doi:10.1186/s13054-014-0643-5)
Supplement: Additional file 3: Table AF2. — Population characteristics. [file 13054_2014_643_MOESM3_ESM.docx]

**Additional File 3**

**Table AF2. Population characteristics**

| **Main author, publication date** |  | **Inclusion criteria** |  | **Exclusion criteria** |  | **Characteristics of patients** | | |  | *P* |
| --- | --- | --- | --- | --- | --- | --- | --- | --- | --- | --- |
|  |  |  |  |  |  | With adverse event |  | Without adverse event |  |  |
| Trunet et al,  1986 |  | All patients |  | Accidental or deliberate self-poisoning |  | n = 97  **mean age (years):** 55.2 years  **sex ratio (M/F):** 0.7  **underlying diseases:**  severe: 76.3% (74)  minor: 19.6% (19)  none: 4.1% (4)  **origin:**  hospital: 55.7% (54)  home: 44.3% (43) |  | ND |  | ND |
| IGICE, 1987 |  | Diagnostic of ADR leading to an ICU admission if no other reason could be found to explain the event |  | ND |  | n = 24  **Origin:**  home: n = 6 (25%)  hospital wards: n = 8 (33%)  radiology: n = 10 (42%) |  | ND |  | ND |
| Nelson et al,  1996 |  | All patients (including overdoses with legal or illegal drugs) admitted in medical ICU,  CCU and internal medicine service, during the studied period |  | ND |  | **median age (years):** 43*  **No drugs prescribed, median [range]:**  3[1-12]*  **ICU LOS days, median [range]:**  4[1-36]*  **Percentage of noncompliant patient:**  65.8%* |  | **median age (years):** 47*  **No drugs prescribed, median [range]:**  2[1-12]*  **ICU LOS days, median [range]:**  6[1-74]*  **Percentage of noncompliant patient:**  15.7%* |  | ND  0.0037  0.0018  < 0.0001 |
| Darchy et al,  1999 |  | All patients admitted in the ICU during the studied period |  | Consensus not reached between evaluators |  | n = 41  **mean age, years ± SD:** 70.8 ± 17,0  **sex ratio (M/F):** 0.41  **ICU LOS days, mean ± SD:** 4.3 ± 2.9  **mean SAPS ± SD:** 14.1 ± 5.0  **No drugs before admission, mean ± SD:**  4,0 ± 2.6  **mortality rate:** 14.6%  **Origin:**  hospital: n = 17 (41%)  home: n = 24 (59%) |  | n = 555  **mean age, years ± SD:** 53.1 ± 20,0  **sex ratio:** 1.38  **ICU LOS days mean ± SD:** 6.0 ±7.5  **mean SAPS ± SD:** 11,1 ± 7.0  **No drugs before admission, mean ± SD:**  2.1 ± 2.2  **mortality rate:** 16.7%  **Origin:** ND |  | < 0.001  < 0.001  0.14  0.008  < 0.001  0.73  ND |
| Hammerman et al,  2000 |  | Major cardiac event (life-threatening problems) |  | Deliberate or accidental self-poisoning  Consensus not reached between evaluators |  | n = 64  **mean age, years ± SD:** 72 ± 8  **sex ratio (M/F):** 0.56 |  | ND |  | ND |

**Table AF2 (continued)**

| **Main author, publication date** |  | **Inclusion criteria** |  | **Exclusion criteria** |  | **Characteristics of patients** | | |  | *P* |
| --- | --- | --- | --- | --- | --- | --- | --- | --- | --- | --- |
|  |  |  |  |  |  | With adverse event |  | Without adverse event |  |  |
| Lehmann et al, 2005 |  | First admission to ICU |  | Readmission to ICU |  | n = 66  **mean age, years ± SD:** 59 **±** 21**  **median ICU LOS days**: 3** |  | ND |  | ND |
| Grenouillet-Delacre et al, 2007 |  | Age > 15 years and 3 months admitted in the medical ICU, and available previous medical history including drugs prescribed in the month before admission |  | **-** Lack of drug treatment data and previous medical history  - Deliberate or unintentional overdose  - Relapse due to non- compliance of self-medication  - Cases judged as doubtful |  | n = 111  **age, years, % (n)**  61-75: 34% (38)  > 75: 32% (36)  **sex ratio (M/F):** 0.50  **ICU LOS days, mean ± SD:** 6.4 ±8  **prescription origin: %** (n)  **hospital**: 55% (61)  **underlying disease: %** (n)  at least one serious disease: 56% (62)  chronic organ failure: 54% (60)  haematological malignancies: 11% (12)  immunodeficiency: 28% (31)  **number of drugs: % (n)**  1-3: 2% (2)  4-6 : 13% (15) ;  > 6: 85% (94)  **reason for admission**  coma: 17% (19)  shock: 22% (25)  **SAPS II:**  < 30: 29% (32)  30-80: 62% (69)  > 80: 9% (10)  **mortality rate:** 19% |  | n = 294  **age, years, % (n)**  61-75: 26% (76)  >75: 19% (55)  **sex ratio (M/F):** 0.57  **ICU LOS days, mean ± SD:** 5.6 ±9  **prescription origin: %** (n)  **hospital**: 33% (98)  **underlying disease: %** (n)  at least one serious disease: 41% (121)  chronic organ failure: 39% (116)  haematological malignancies: 2% (6)  immunodeficiency: 16% (48)  **number of drugs: % (n)**  1-3: 19% (55)  4-6: 22% (64) ;  > 6: 59% (175)  **reason for admission**  coma : 29% (86)  shock: 14% (43)  **SAPS II:**  < 30: 44% (131)  30-80: 46% (136)  > 80: 9% (27)  **mortality rate:** 17% |  | 0.02  <0.001  0.18  0.45  <0.001  0.01  0.01  0.001  0.01  0.02  <0.001  0.01  0.02  0.003  0.32  0.71 |
| Rivkin et al,  2007 |  | All patients admitted to the medical ICU during the study period |  | - Intentional overdoses  - ADR occurring during the medical ICU stay  - Patients admitted over the weekends |  | n = 21  **ICU LOS days, mean [range]:** 5.7 [0.5-35] |  | ND |  | ND |

**Table AF2 (continued)**

| **Main author, publication date** |  | **Inclusion criteria** |  | **Exclusion criteria** |  | **Characteristics of patients** | | |  | *P* |
| --- | --- | --- | --- | --- | --- | --- | --- | --- | --- | --- |
|  |  |  |  |  |  | With Adverse event |  | Without Adverse event |  |  |
| Schwake et al,  2009 |  | All patients aged 14 and above |  | ADR classified as possible, unlikely and doubtful |  | n = 99  **median age, years, (IQR):** 71 (62-79)  **sex ratio (M/F):** 1,06  **median SAPS II (IQR):** 37 (32-42)  **ICU LOS days, mean ± SD:** 2.3 ± 2.0  **hospital mortality% (n):** 2% (2)  **six-month mortality% (n):** 9% (9)  **origin:**  from home: 82.8% (82) ;  from hospital: 17.2% (17) |  | ND |  | ND |
| Mercier et al, 2010 |  | All patients first admitted in the ICU during the studied period |  | - Readmissions to the ICU  - IE considered as possible or unlikely |  | Not specifically documented for patients with ADE  n = 103 patients with IE***  **mean age, years ± SD:** 59.6 ± 17.9***  **sex ratio (M/F):** 1.34***  **underlying disease:**  metabolic: 38.8% (40)***  hematologic and cancer: 21.4% (22)***  **Mac Cabe Score***:**  0: 46.6% (48)***  1: 41.7% (43)***  2: 11.7 %(12)***  **No physicians involved, median [range]:**  3[1-6]***  **No drugs prescribed, median [range]:**  7[1-21]***  **mortality rate:** 11.3%*** |  | n = 425 patients without IE  **mean age, years ± SD:** 55.1 ± 20.0  **sex ratio (M/F):** 1,28  **underlying disease:**  metabolic: 27.5% (117)  hematologic and cancer: 7.5% (32)  **Mac Cabe Score:**  0: 60.1% (255)  1: 32.6% (138)  2: 7.3 % (31)  **No physicians involved, median [range]:** 2[0-8]  **No drugs prescribed, median [range]:**  4[0-18]  **mortality rate:** 15.5% |  | 0.05  0.85  0.03  <0.001  0.03  <0.001  <0.001  0.27 |
| Nazer et al, 2012 |  | All patients first admitted in the ICU during the studied period |  | - ADE associated with blood products  - consensus not reached between evaluators  **-** drug overdose or poisoning |  | n = 57  **mean age, years ± SD:** 50,4 ± 16.1  **sex ratio** (M/F)**:** 1.11  **cancer type% (n):**  **haematological malignancies:** 56.1% (32)  **LOS, mean ± SD:** 6.2 ± 9.8  **mortality rate:** 28.1% |  | n = 192  **mean age, years ± SD:** 52.4 ± 15,6  **sex ratio** (M/F)**:** 1.23  **cancer type% (n):**  **haematological malignancies:** 19.8% (38)  **LOS, mean ± SD:** 6.2 ± 8  **mortality rate:** 31.3% |  | 0.41  0.68  <0.0001  0.99  0.54 |

Abbreviations used: ADE, Adverse Drug Event; ADR, Adverse Drug Reaction; CCU, Coronary Care Unit; ICU, Intensive Care Unit; IE, Iatrogenic Event; IGICE, Italian Group on Intensive Care Evaluation; IQR, Inter Quartile Range; LOS, Length Of Stay; M/F, Male/Female; ND, not documented; No, number of; SAPS II, Simplified Acute Physiology Score II; SD, Standard Deviation.

*Not specifically documented: pooled result of the 3 studied departments including the department of internal medicine.

**Not specifically documented for patients with adverse drug events, calculated among patients with iatrogenic medical event

***Not specifically documented for patients with adverse drug events, also includes patients with events related to procedural complications.
