# Supplementary material for: A systematic review of adult admissions to ICUs related to adverse drug events
Source: Crit Care. 2014 Nov 25;18(6):643. doi: 10.1186/s13054-014-0643-5 (PMC4422001; doi:10.1186/s13054-014-0643-5)
Supplement: Additional file 4: Table AF3. — Clarification of event terminology and incidence of event-related admissions in the ICU. [file 13054_2014_643_MOESM4_ESM.docx]

**Additional File 4**

**Table AF3. Clarification of event terminology and incidence of event-related admissions in the intensive care unit**

| **Main author, publication date** |  | **Main outcome(s)/ definition** |  | **Main outcome(s) considered as: ADE, ADR or medication errors** |  | **Incidence of event-related admissions in the ICU** |
| --- | --- | --- | --- | --- | --- | --- |
| Trunet et al, 1986 |  | Drug-induced illness: disease that results from drug administration for prophylaxis, diagnosis or therapy  Adverse Drug Reaction: [[1](#_ENREF_1)]  Therapeutic Errors: over dosage, inappropriate route of administration and prescribing in spite of contra-indication |  | Drug-induced illness: ADE  Adverse Drug Reaction: ADR  Therapeutic Errors: medication errors |  | ADE: 97 /1651 admissions **= 5.9%**  ADR: 66/1651 admissions **= 4.0%**  medication errors: 31/1651 admissions **= 1.9%** |
| IGICE, 1987 |  | Adverse Drug Reaction (ADR): Not defined |  | ADR |  | ADR: 24/4537 = **0.53%** |
| Nelson et al, 1996 |  | Adverse Drug Reaction (ADR): Not defined |  | ADR |  | ADR: 21 /127 admissions = **16.5%** |
| Darchy et al, 1999 |  | Iatrogenic Disease: disease induced by a drug prescribed by a physician, or after medical or surgical procedure, excluding intentional overdose, nonmedical intervention, or unauthorized prescription, and environmental events (falls, equipment defects) |  | Iatrogenic Disease: ADE |  | ADE: 41/623 admissions = **6.6%** |
| Hammerman et al, 2000 |  | Iatrogenic Event: any adverse event that resulted from therapy |  | Iatrogenic Event: ADE |  | ADE: 64/ 2559 admissions = **2.5%** |
| Lehmann et al, 2005 |  | Adverse Drug Event: an injury resulting from complications of “non-invasive therapy” (the latter term was not defined) |  | ADE |  | ADE: 21/5727 admissions = **0.37%** |
| Grenouillet-Delacre et al, 2007 |  | Life-threatening Adverse Drug Reaction: Serious ADR [[2-4](#_ENREF_2)] that resulted in acute organ failure leading to admission, in the intensive care unit, in artificial life support, or in death |  | ADR |  | ADR: 132 among 111 patients/405 admissions = **27.4%** |
| Rivkin et al, 2007 |  | Adverse Drug Reactions (ADR): any noxious, unintended or undesired effect of a drug occurring at dosages administered in humans for prophylaxis, diagnosis or treatment [[5](#_ENREF_5)] |  | ADR |  | ADR: 21/281 admissions = **7.5%** |
| Schwake et al, 2009 |  | Adverse Drug Reactions (ADR): defined according to WHO classification system [[6-9](#_ENREF_6)] |  | ADR |  | ADR: 99 /1554 admissions = **6.4%** |
| Mercier et al, 2010 |  | Adverse Drug Event: not defined |  | ADE |  | ADE: 50/528 admissions = **9.5%** |
| Nazer et al, 2012 |  | Adverse Drug Event (ADE): injury or harm resulting from medical intervention related to a drug [[10](#_ENREF_10)] |  | ADE |  | ADE: 57/249 admissions = **22.9%** |

Abbreviations used: ADE, Adverse Drug Event; ADR, Adverse Drug Reaction; IGICE, Italian Group on Intensive Care Evaluation; ND, Not Documented; WHO, World Health Organization.

**REFERENCES**

1. Karch FE, Lasagna L: **Adverse drug reactions. A critical review**. *JAMA* 1975, **234**(12):1236-1241.

2. Edwards IR, Aronson JK: **Adverse drug reactions: definitions, diagnosis, and management**. *Lancet* 2000, **356**(9237):1255-1259.

3. Roswell R, Van Diepen LR, Jones JK, Hicks WE: **Adverse drug reactions**. *Lancet* 2001, **357**(9255):561-562.

4. Nebeker JR, Barach P, Samore MH: **Clarifying adverse drug events: a clinician's guide to terminology, documentation, and reporting**. *Ann Intern Med* 2004, **140**(10):795-801.

5. **WHO draft guidelines for adverse event reporting and learning system** [http://www.who.int/patientsafety/events/05/Reporting_Guidelines.pdf]

6. Lazarou J, Pomeranz BH, Corey PN: **Incidence of adverse drug reactions in hospitalized patients: a meta-analysis of prospective studies**. *JAMA* 1998, **279**(15):1200-1205.

7. Grenouillet-Delacre M, Verdoux H, Moore N, Haramburu F, Miremont-Salame G, Etienne G, Robinson P, Gruson D, Hilbert G, Gabinski C *et al*: **Life-threatening adverse drug reactions at admission to medical intensive care: a prospective study in a teaching hospital**. *Intensive Care Med* 2007, **33**(12):2150-2157.

8. Somers A, Petrovic M, Robays H, Bogaert M: **Reporting adverse drug reactions on a geriatric ward: a pilot project**. *Eur J Clin Pharmacol* 2003, **58**(10):707-714.

9. Ramesh M, Pandit J, Parthasarathi G: **Adverse drug reactions in a south Indian hospital--their severity and cost involved**. *Pharmacoepidemiol Drug Saf* 2003, **12**(8):687-692.

10. Bates DW, Cullen DJ, Laird N, Petersen LA, Small SD, Servi D, Laffel G, Sweitzer BJ, Shea BF, Hallisey R *et al*: **Incidence of adverse drug events and potential adverse drug events. Implications for prevention. ADE Prevention Study Group**. *JAMA* 1995, **274**(1):29-34.
